# Living Arrangements and Dementia Care Networks: Variations in Size, Composition, and Care Tasks

**DOI:** 10.1002/alz70858_097451

**Published:** 2025-12-24

**Authors:** Natasha L Nemmers, Wenjing Li, Wen‐hua Lai, Amanda N Leggett

**Affiliations:** ^1^ Wayne State University, Detroit, MI, USA; ^2^ Wayne State University Institute of Gerontology, Detroit, MI, USA

## Abstract

**Background:**

Approximately one in four older Americans with cognitive impairment or dementia lives alone, but there is limited understanding of who provides their care and how their care is structured. Current research often relies on binary classifications of living alone or with others, which miss critical nuances. Among multi‐person households, living with a spouse versus living with other cohabitants can distinctly affect caregiving dynamics. This study examines how dementia care networks differ based on these living arrangements.

**Methods:**

We utilized data from the 2022 National Health and Aging Trends Study, categorizing older adults with possible or probable dementia by living situation: living alone (*N* = 265), spouse present (*N* = 369), or with others only (*N* = 328). We analyzed caregiving network size, relationship composition, and care tasks, employing chi‐square tests and ANOVAs for comparisons, with sensitivity analyses for non‐dementia groups.

**Results:**

Individuals living alone with dementia had significantly more helpers (*M* = 1.92, similar to those living with others, *M* = 1.88, *p* = .127) compared to those living with a spouse (*M* = 1.48, *p* < .001). Despite larger networks, individuals living alone with dementia had significantly less help across care domains such as household, medical, and transportation than other groups (*p*s< .001), except mobility and self‐care (living alone v. spouse, *p* = .448). In terms of relationship composition, the presence of a child caregiver was similar across living arrangements. However, living alone was associated with a higher number of non‐family caregivers (e.g., paid helpers or friends) compared to those living with a spouse (*p* < .001). The presence of any non‐immediate and non‐family member as a caregiver was notably lower for those living with a spouse versus alone or with others (*p*s< .001).

**Conclusion:**

The findings reveal that while those living alone with dementia have larger networks, these helpers, often including non‐kin, provide less practical support, indicating potential unmet needs. In contrast, spousal caregivers shoulder a substantial care burden with limited additional support. Diversified networks in multi‐person households distribute care but may complexify coordination. These insights underscore the need for tailored interventions to support caregivers and enhance care for older adults based on living arrangements. Future research should investigate how these network dynamics influence health outcomes and service utilization.

